# Exploring community reintegration among Nigerian stroke survivors

**DOI:** 10.4102/sajp.v79i1.1857

**Published:** 2023-06-21

**Authors:** Michael O. Ogunlana, Olufemi O. Oyewole, Abiola Fafolahan, Pragashnie Govender

**Affiliations:** 1 Department of Physiotherapy, Federal Medical Centre Abeokuta, Abeokuta, Ogun State, Nigeria; 2 Department of Occupational Therapy, College of Health Sciences, University of KwaZulu-Natal, Durban, South Africa; 3 Department of Physiotherapy, Olabisi Onabanjo University Teaching Hospital, Sagamu, Nigeria

**Keywords:** community reintegration, stroke survivors, rehabilitation, Nigeria, enablers, barriers

## Abstract

**Background:**

Community reintegration is one of the ultimate goals of stroke rehabilitation. The increasing burden of stroke morbidity by other non-communicable diseases in Nigeria indicated the need for our study.

**Objectives:**

The authors explored the factors contributing to successful community reintegration among Nigerian stroke survivors.

**Method:**

We conducted an explorative qualitative study design to achieve this aim using in-depth semi-structured interviews with 12-purposively sampled stroke survivors.

**Results:**

Three overarching themes emerged: restriction of participation experienced by stroke survivors, activity limitation as pointers to the quality-of-life experience of stroke survivors and enablers or barriers to community reintegration for stroke survivors. Among the core, sub-themes included incapability of returning to work, difficulty performing domestic activities, social isolation or separation, recreation and leisure time. Enablers of community reintegration included creating a positive mindset, encouragement and social support, while barriers included mobility and speech or language challenges.

**Conclusion:**

Stroke survivors have challenges in returning to work and experience varying levels of activity limitation, which affects their quality of life with identifiable enablers or barriers to community reintegration.

**Clinical implications:**

Stroke survivors with severe functional deficits should be monitored closely and given further rehabilitative assistance to aid functional recovery, thereby facilitating community reintegration.

## Introduction

Stroke is the second most common cause of death and disability worldwide (Katan & Luft 2018). The burden of stroke has substantially increased over the past few decades as a result of astronomical population growth and ageing with the increased prevalence and incidence of stroke-modifiable risk factors common in low- and middle-income countries (Katan & Luft 2018). The incidence of stroke in Africa ranges from 25 per 100 000 persons in Lagos, Nigeria to 250 per 100 000 persons in Al-kharga, Egypt, while the prevalence of stroke ranges from 15 per 100 000 in Ethiopia to 963 per 100 000 in Egypt and 1460 per 100 000 in the Niger delta regions of Nigeria (Owolabi et al. 2018). The annual incidence rate of stroke in Africa is up to 316 per 100 000 individuals, and the prevalence rate of 1460 per 100 000 reported from one region of Nigeria is clearly among the highest in the world (Akinyemi et al. 2021). Increasing knowledge in the medical management of stroke emergencies results in a gradual increase in stroke survivors with stroke-related morbidities. Dementia and depression are morbidities that are confirmed consequences of stroke (Filipska et al. 2020). These morbidities have necessitated increased utilisation of rehabilitation facilities.

Neurological rehabilitation is usually a lengthy process that requires prolonged treatment periods both in inpatient and outpatient settings producing relevant benefits in terms of functional improvement, fewer unnecessary complications and better coordination of services (Barnes et al. 2004). Stroke rehabilitation is a subset of neurological rehabilitation that greatly depends on the structures of care and processes of care (Teasell et al. 2009). Care processes such as early admission of patients to rehabilitation, higher therapy intensities, task-specific therapies and careful discharge planning designed to meet the patient’s specific needs are vital for better outcomes (Teasell et al. 2009). Stroke rehabilitation is continued at the home or community level to customise the intervention to each survivor’s natural environment. Stroke rehabilitation services are more cost-effective when delivered at the community level than outpatient care (Allen et al. 2019; Godwin, Wasserman & Ostwald 2011). Community-based neurorehabilitation for Nigerians with a stroke incident may not be readily available in the home environment as care delivery is mainly outpatient or inpatient. This may inevitably negatively affect the functional recovery of stroke patients with a resultant consequence on the community reintegration of stroke survivors.

Community reintegration is one of the primary goals of rehabilitation for stroke survivors and is threatened by the individuals’ lack of resumption of previous roles and daily activities (Palmer & Glass 2003). When rehabilitation services are inaccessible, community reintegration is thus compromised. Visagie and Schneider indicated that individuals with disabilities have limited access to rehabilitation, which negatively influences the individuals’ ability to reintegrate into the community (Visagie & Schneider 2014). Esbjörnsson et al. highlighted that being proficient at participating in meaningful areas of their life, such as work, independent living, leisure and social roles, is the essential component required for the community (Esbjörnsson, Skoglund & Sunnerhagen 2013). Given the increasing burden of stroke morbidities that are occasioned by the presence of other non-communicable diseases in our locality and Nigeria, there is a need to explore the factors that contribute to successful community reintegration and how rehabilitation could aid in this process among Nigerian stroke survivors. The outcome of our study may make a case for the standardisation and institutionalisation of home assessment, visits and intervention by rehabilitation professionals.

## Methods

An explorative qualitative study design was used for data collection through an in-depth semi-structured interview with stroke survivors to explore their community reintegration.

Our study was conducted in the Abeokuta Metropolis. Abeokuta town, capital of Ogun State, southwestern Nigeria. A tertiary health facility in Abeokuta that hosts a stroke multidisciplinary team and a stroke survivors club was the focal point for our study. Stroke survivors who received acute stroke care were traced to their homes after discharge and invited to participate in our study after their informed consent was obtained.

### Study population and sampling strategy

The target population included persons who had suffered a stroke (12–24 weeks post-discharge from acute stroke care) and currently residing in the Abeokuta metropolis of Ogun State, Nigeria. The stroke survivors were purposively sampled to participate in our study. The stroke admission registers of Federal Medical Centre Abeokuta Stroke Multi-Disciplinary Team (FMCASMDT) and Stroke Survivors support, and rehabilitation club of Nigeria (SSSRCN) were used in identifying stroke survivors’ residents in Abeokuta metropolis who met the inclusion criteria for participation. Included participants were able to understand and speak (communicate) either Yoruba or English, they had an episode of a stroke incident at least 3 months before the interview with a current score of not more than 3 out of 6 on the Modified Rankin Scale (Wilson et al. 2002) and they were over the age of 18 years. Any person with a severely impaired level of functioning before the stroke was excluded. The participants who gave consent to participate, having met the inclusion criteria, were included.

### Procedure for data collection and analysis

Following recruitment, time and location for the interviews were negotiated. The authors explained the purpose of our study, and participants were reassured that participation was voluntary and that they could withdraw from our study at any time, with confidentiality maintained. Data were collected using semi-structured interviews that spanned from 45 min to an hour. Participants completed a proforma that obtained socio-demographic and stroke-related information before the main interview. This information included age, gender, date of stroke, affected side, duration of hospitalisation, whether rehabilitation was received, the frequency, and if it was continuing, educational level, and employment status before and after the stroke. The semi-structured interview questions were open-ended to ensure that participants expressed their views and shared their experiences about factors and resources that hinder or aid their quality of life (QoL) and community reintegration. The International Classification of Functioning, Disability and Health (ICF) model, literature and our study’s aims and objectives guided the development of the interview schedule. Interviews were audio-recorded and written field notes were generated during the process. The interviews were translated, transcribed and analysed using the framework approach (Gale et al. 2013), which identified codes, categories and themes. Analysis occurred before the ensuing interview to ensure that sampling ceased once data redundancy was reached.

### Trustworthiness and credibility

Credibility was guaranteed by utilising thick descriptions and triangulation (Anney 2014). A thick description was possible by enquiring about the context and situation of the participants (from the demographic proforma). Adequate information on the study context was obtained to ensure transferability.

## Findings

**FIGURE 1:**
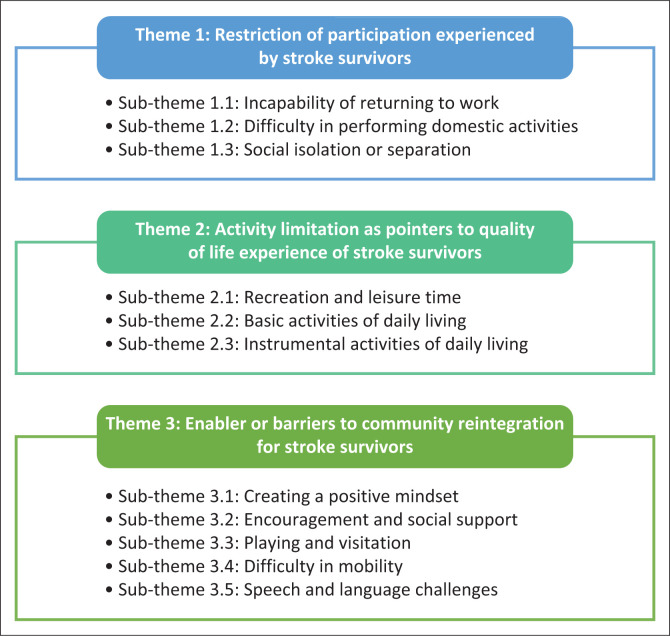
An overview of themes and sub-themes.

Table 1 presents the participants’ characteristics with the first stroke incidence. About 58% were females, and 75% had at least a secondary education. The stroke survivors were 56.8 ± 8.5 years old and had an average of 12 days of hospital admission and/or stay. They were interviewed within 9 months post-stroke. Various themes and sub-themes emerged from this study (Figure 1). The qualitative results (verbatim spoken word of participants) are presented in italics.

**TABLE 1:** Characteristics of the stroke survivors.

Unique identifier (Patients)	Gender	Age	Level of education	Stroke type	Stroke laterality	Length of stay	Time since stroke incident	Work before stroke	Work after stroke
P1	M	51	SSCE	Ischaemic	Left	A month and 2 weeks	3 months	Driver	No
P2	F	52	SSCE	Haemorrhagic	Right	4 days	6 months	Sells foodstuff	No
P3	F	69	No formal	Ischaemic	Left	Not admitted	13 months	Petty trader	No
P4	M	62	BSc	Ischaemic	Right	4 days	4 months	Counsellor	Yes
P5	M	56	BSc	Ischaemic	Left	5 days	1 month	Specialist teacher	Yes
P6	F	64	Elementary	Ischaemic	Right	10 days	Three quarters of a month	Retired civil servant	Yes
P7	F	62	SSCE	Ischaemic	Right	3 weeks	2 months	Trader, fashion designer	No
P8	M	51	SSCE	Ischaemic	Left	10 days	3 months	Driver	No
P9	F	38	SSCE	Haemorrhagic	Left	2 weeks	2 months	Factory worker	No
P10	F	66	SSCE	Ischaemic	Right	3 weeks	6 months	Trader	No
P11	M	57	SSCE	Ischaemic	Right	2 weeks	4 months	Motor part dealer	Yes
P12	F	53	Primary	Ischaemic	left	Home care	3 months	Foodstuff trader	No

### Theme 1: Restriction of participation experienced by stroke survivors

#### Sub-theme 1.1: Incapability of returning to work

Returning to pre-stroke occupation is an essential outcome of successful community reintegration. Returning to work is a fundamental goal for many stroke survivors. Thus, stroke survivors were prompted on whether they were able to engage in any productive occupation following the stroke incident. About 67% of stroke survivors could not return to their premorbid work because of stroke-associated pain, movement impairment and low energy or strength level. Some of the expressions included:

‘Before the stroke, I wake up by 6 am, bath and go to the office but since this stroke, I have not even attended work at all.’ (P5, male, 56 years old)

P5 is a 56-year-old male teacher who was near retirement from office work; since the stroke incident, he had been unable to return to his routine of waking up at 6 am to perform his basic and instrumental activities of daily living. For P5 to reintegrate into the community, he might need to attain mobility ability that is near to his premorbid state. P7, P1 and P3 were unable to return to work because of limb muscle weakness that hindered the performance of activities of daily living. Weakness of lower limb muscles hindered P7 from returning to her tailoring job:

‘I can’t sew because of my leg, I only cut the cloth and my children sew for me.’ (P7, female, 62 years old)

P1 also had lower limb and upper limb muscle impairment that hindered him from safely driving a car. He has been unable to return to this instrumental activity of daily living since the occurrence of the stroke but for P3, the reason for his non-returning to work was because of low energy levels that make him easily fatigued:

‘I was driving but since August I haven’t been able to do it … I can’t move like before. I can’t run to do things.’ (P1, male, 51 years old)

‘I was hardworking before the stroke. I can’t do anything now. I can’t go to the market anymore … This stroke stopped me from working. I don’t have much energy now … because I don’t have strength.’ (P3, female, 69 years old)

It is clear that the consequence of a stroke is muscle impairment that hinders the activity of daily living that can further cause participation restriction.

**Sub-theme 1.2: Difficulty in performing domestic activities:** Engaging in domestic chores is an expected activity that stroke survivors seek. Restoration of these activities was considered essential for improved QoL. These activities are instrumental to community reintegration and sometimes a source of livelihood. P2 and P9 were unable to prepare their meals, while P2 reported that this disability was associated with pain and inability to lift the affected limb. P4 is a retired counsellor who runs a beer parlour, and he is involved in sweeping the office and loading crates of drinks. He could not participate in this work since the stroke event. Some quotes that contained these claims are:

‘I try to cook but the pain is too much, sometimes I wake up and can’t lift my leg.’ (P2, female, 52 years old)

‘I can’t cook again. I can’t do anything again.’ (P9, female, 38 years old)

‘I used to do what I cannot do again as a bar attendant, I should be up to sweep, load beer and other drinks but I don’t do them again. Sometimes, I just sit there looking at them.’ (P4, male, 62 years old)

**Sub-theme 1.3: Social isolation or separation:** Most participants described living sedentary lifestyles and limited social and religious engagements compared with their premorbid states. Social isolation was associated with the movement challenges sequel to the stroke incident and housing arrangements that worsen mobility like living in a one or two-storey building that does not have an elevator:

‘My sister died; I couldn’t go because of this stroke.’ (P4, male, 62 years old)

‘I don’t go out of my compound, but I move around the house. From the bedroom to the parlour and other places in the house.’ (P6, female, 64 years old)

‘No, but I can’t go out. Our house is upstairs. I stay indoors, I don’t go out.’ (P9, female, 38 years old)

Some stroke survivors indicated feelings of being unoccupied and/or bored. Their expressions included:

‘It makes me idle. I find myself sleeping or just gossiping.’ (P6, female, 64 years old)

‘I don’t like life after the stroke. It is very boring; I just sit down and rest.’ (P7, female, 62 years old)

‘I bath, I sleep, listen to the radio.’ (P8, male, 51 years old)

‘I do nothing; I sleep, cook, and move around the house.’ (P12, female, 53 years old)

Many of the stroke survivors expressed their inability to perform religious activities:

‘It has affected me from going to church.’ (P1, male, 51 years old)

‘After the stroke, I haven’t gone to church.’ (P4, male, 62 years old)

‘Before I go to church on Sundays and Thursdays and I still go to church, but I only go on Sundays.’ (P9, female, 38 years old)

‘Before I preach in church as a pastor and I do so many things but since this stroke has started, I have not been attending church.’ (P5, male, 56 years old)

‘I still pray at home; I can’t go to the mosque. Praying is usually more interesting with others. It is boring when I do it alone.’ (P12, female, 53 years old)

#### Theme 2: Activity limitation as pointers to the quality-of-life experience of stroke survivors

One of the critical outcomes in stroke management is QoL. An aspect of recreation and leisure time, basic and instrumental activities of daily living were revealed as cynosures of QoL for the stroke survivors.

**Sub-theme 2.1: Recreation and leisure time:** Most of the stroke survivors were able to engage in recreation and leisure time activities such as watching television with an emphasis on movies and football matches, singing, ‘cracking’ jokes. The satisfaction associated with these leisure time activities appears to reflect the QoL of stroke survivors:

‘I watch movies, I gist. I stay on the balcony to see what is going on outside.’ (P9, female, 38 years old)

‘I sing songs that I like, and it makes me feel happy.’ (P9, female, 38 years old)

‘I crack jokes. But I can’t do that now because of my speech. The new hobby I have is talking with people around me, they make me talk. I enjoy companionship now. Before I was a drinker. I like merriment and I like to give.’ (P4, male, 62 years old)

‘I watch movies. I sleep after watching and it makes me feel okay.’ (P2, female, 52 years old)

**Sub-theme 2.2: Basic activities of daily living:** Many of the stroke survivors were able to perform basic activities of daily living. They expressed their independence. They dress, bathe, cook and eat themselves. Some are able to achieve independence by gradual restoration of function:

‘I go to the toilet, bathe, and do some other things by myself without support.’ (P7, female, 62 years old)

‘I cook just my food sometimes too. I don’t want my hand to be stiff. I eat by myself.’ (P2, female, 52 years old)

‘I wear cloth by myself.’ (P1, male, 51 years old)

‘I dress myself before it was my daughter. I told her to stop so I am forcing myself.’ (P2, female, 52 years old)

While some are able to perform basic activities of daily living through modification of such activities like sitting while having a bath and use of human support:

‘I bath by myself and go to the toilet myself though they fetch water for me.’ (P11, male, 57 years old)

‘I bath myself but I sit on a chair while bathing.’ (P3, female, 69 years old)

‘I eat [*by*] myself though with the left hand.’ (P6, female, 64 years old)

**Sub-theme 2.3: Instrumental activities of daily living:** Many of the participants can prepare meals, engage in shopping tasks, independently drive, and communicate effectively:

‘I can cook for myself too. For instance, I can make eba [*boiled cassava flakes*] for myself alone. Not in large quantity.’ (P3, female, 69 years old)

‘I can drive a small car but not a big one like a truck.’ (P1, male, 51 years old)

However, few stroke survivors reported they are unable to execute community survival tasks such as shopping or frequenting the market:

‘I can’t go to the market. Before I would have gone to the market as early as 6 am.’ (P2, female, 52 years old)

#### Theme 3: Enablers/barriers to community reintegration for stroke survivors

A few themes that emerged as enablers of community integration include a positive mindset, encouragement and social support, playing and visitation.

**Sub-theme 3.1: Creating a positive mindset:** Many stroke survivors have a positive mindset, and this helps a lot in community integration. They express:

‘I am happy that I am still alive. Many people have died. I thank God I am alive.’ (P2, female, 52 years old)

‘I thank God that I am alive, some people have died even though they didn’t face something like this.’ (P9, female, 38 years old)

‘I am not worried. I am praying to God to heal me.’ (P2, female, 52 years old)

‘I am satisfied. I continue my life.’ (P5, male, 56 years old)

‘I am trying though it is not easy, but I don’t want my body to be stiff so, I try to do my best. I have been seeing changes when I started coming to the hospital.’ (P12, female, 53 years old)

**Sub-theme 3.2: Encouragement and social support:** Many participants express encouragement from family members and society, as well as financial and family support:

‘My children, friends and family encourage me that I will be okay again.’ (P2, female, 52 years old)

‘Yes, my children and daughter-in-law take care of me. They didn’t allow me to feel anything.’ (P3, female, 69 years old)

‘Yes, my children cook for me, joke with me and do so many things for me and it makes me happy.’ (P10, female, 66 years old)

‘They support me very well. My husband and his sister. My husband’s sister cooks for me.’ (P9, female, 38 years old)

‘Yes. I have support from my children and my husband.’ (P6, female, 64 years old)

‘They give me food, bath for me, and help me go to the toilet.’ (P5, male, 56 years old)

‘I get support from the church too. My pastor brought me gifts.’ (P4, male, 62 years old)

‘My friends. Sometimes, they give me cash.’ (P11, male, 57 years old)

‘They give me food, they give me money, etc. They sponsor my hospital bills.’ (P7, female, 62 years old)

**Sub-theme 3.3: Playing and visitation:** Playing and visitation also facilitate community reintegration among stroke survivors as expressed by them:

‘I play with my younger sister’s children. My church members come to visit too, and they encourage me.’ (P2, female, 52 years old)

‘They crack jokes with me, we laugh together. I am well taken care of.’ (P6, female, 64 years old)

‘They come to visit me.’ (P9, female, 38 years old)

‘Some other people also come to visit me.’ (P10)

**Sub-theme 3.4: Difficulty in mobility:** However, stroke survivors identified barriers to community reintegration as challenges of mobility. As earlier stated, these impairments made functional restoration complex or impossible:

‘I cannot move like I used to.’ (P6, female, 64 years old)

‘I can’t walk well like before.’ (P11, male, 57 years old)

‘I hold things when I am going about the house even when I am going to the toilet.’ (P9, female, 38 years old)

‘It is not easy to move about. The (bus) conductor had to help me when I was coming to the hospital today.’ (P12, female, 53 years old)

**Sub-theme 3.5: Speech and language challenges:** The participants clearly expressed the debilitating effects of speech and language challenges presented by stroke survivors. Some of these participation restrictions experienced by the survivors are described in the verbatim quotes below:

‘I find it difficult to say some words, yes. It is hard for me to explain where I am going to people.’ (P4, male, 62 years old)

‘I can’t talk the way I used to talk. My tongue was twisted initially. I couldn’t talk well initially but I am getting better. I can talk better now but I can’t shout.’ (P6, female, 64 years old)

‘A lot, I can’t answer calls correctly. Whatever the person on the phone says, I will just be listening. It is hard for me to reply, and it gets me annoyed. Somebody told me to go to see a brain specialist to give me drugs.’ (P4, male, 62 years old)

## Discussion

Our study reports the experience of community reintegration of Nigerian stroke survivors. Three themes and 11 sub-themes were identified. Most of the stroke survivors reported an inability to return to work. The findings reveal return to work as an important factor in community reintegration. When stroke survivors can perform their instrumental activities of daily living, which may enhance their potential for gainful employment through meaningful occupation; community reintegration may be ensured. Return to work is a well-sought-after goal among stroke survivors, especially those of working age. Returning to work does not only improve brain health and cognitive skills but also provides an opportunity to socialise, thereby enhancing their QoL (Scott et al. 2019). Studies have reported that returning to work post-stroke is difficult but possible over a longer period (Olaoye, Soeker & Anthea 2021; Palstam et al. 2019; Westerlind, Persson & Sunnerhagen 2017; Westerlind et al. 2020; Yousef et al. 2020). These studies report a varied proportion (29% – 60%) of stroke survivors who do return to employment. About one-third of stroke survivors in our study reported returning to work. The ability to return to work after a stroke is influenced by complex factors ranging from physical, social, and cognitive to environmental factors (Ashley, Lee & Heaton 2019; Brannigan et al. 2017; Jellema et al. 2017). Some of these factors require consideration during and following inpatient rehabilitation to facilitate a successful return to work among stroke survivors. There is, therefore, a need to provide community support services to optimise the process of disability management and return to work (Brannigan et al. 2017; Soeker & Olaoye 2017). A meta-synthesis of qualitative research on return to work among stroke survivors emphasise the principles of adaptiveness, purposefulness, and cooperativeness for optimal reintegration into the workplace (Schwarz, Claros-Salinas & Streibelt 2018).

One of the physical factors that encourages community reintegration is community mobility, which is determined by walking speed or gait speed. Mulder et al. and Mwaka-Rutare et al. report that a stroke survivor needed a gait speed ranging between 0.5 and 0.8 metres per second to be community ambulatory (Mulder et al. 2019; Mwaka-Rutare et al. 2020). Although the gait speed of stroke survivors in our study was not objectively assessed, the survivors mainly reported that their inability to walk functionally restricted their participation in all activities. Difficulty in mobility is the main hindrance in returning to work and community reintegration.

Stroke survivors reported difficulty in performing domestic activities such as cooking. A stroke often causes profound disruption in domestic activities, loss of control, independence and confidence (Van Dongen et al. 2021). These may lead to frustration and negative emotions (Theeke et al. 2017). Therefore, a programme to improve impairments and optimise function must be targeted during rehabilitation. As a result of limited domestic activity, stroke survivors have a feeling of being unoccupied. This is compounded by social isolation or separation reported by many participants in our study. Most participants could not return to work or attend religious gatherings and social functions. These activity limitations and participation restrictions may lead to role reversal and negative emotions. Stroke survivors often attach value to social interaction; even a task such as sitting down and talking to family members or relatives has resonated in the literature (Brookfield & Mead 2016; Govender et al. 2019; Theeke et al. 2017; Van Dongen et al. 2021).

Most stroke survivors experienced varying levels of QoL in terms of recreation and leisure time, as basic and instrumental activities of daily living. They appeared to be compelled to engage in leisure activities such as watching movies, television and singing songs because they had to get busy because they had more time post-stroke to invest in leisure. However, their performance of their basic and instrumental activities of daily living signifies a level of functioning post-stroke and engaging in these activities has been shown to influence the QoL positively (Van Dongen et al. 2021). A qualitative study has explained QoL in terms of the reconstruction of the embodied self, which follows three intertwined processes: a familiar self, an unfamiliar self, and a recovery of self (Pedersen et al. 2019). The more the stroke survivors see their familiar self (I can or can’t), unfamiliar self (person before and after stroke) and recovery of self (acceptance, progress, adjustments and management in life); the more their QoL is affected. Therefore, QoL is related to progress in functional recovery or adjustments for engaging in meaningful activities in life (Pedersen et al. 2019). Pedersen et al. further explained that ‘reconstruction of the embodied self can be understood as an ongoing and interrelated process of being, doing, belonging and becoming’ (Pedersen et al. 2019). Our study and others demonstrate that successful return to work, resumption of activities, presence of professional support and good social relations during the recovery process enhance stroke survivors’ QoL (Brookfield & Mead 2016; Ghanbari Ghoshchi et al. 2020; Hebblethwaite & Curley 2015; Pedersen et al. 2019; Theeke et al. 2017; Van Dongen et al. 2021). Therefore, return to work should be a major goal after symptom stabilisation to ensure optimal QoL restoration among stroke survivors.

The positive mindset exhibited by some stroke survivors may be responsible for their recovery, which may enhance community reintegration. Our participants are from a deeply religious and cultural society that encourages a positive outlook under all circumstances. Some survivors were grateful to God that they were alive; others were happy to be alive and some expressed life satisfaction. This happiness stems from their nature of perseverance and ease of acceptance of the stroke and its sequels. Acceptance and perseverance are key factors in developing a positive mindset (Theeke et al. 2017). The authors suggest that a positive psychological outlook to actively participate in physical, mental, emotional and psychological recovery is required for stroke survivors’ successful recovery (Theeke et al. 2017). Thus, stroke survivors’ adaptability, perseverance and ability to overcome emotional challenges can expedite community reintegration (Walsh et al. 2015). As noticed before that social support received from the families, friends, neighbours and churches by stroke survivors serves as an enabler of their community reintegration (Govender et al. 2019). The stroke survivors in our study corroborate this observation as they expressed social support enjoined as helping them in their reintegration into the community. This may be a factor helping in achieving improved QoL as suggested by our data. Strong social support has been linked with an increase in QoL among stroke survivors (Govender et al. 2019; Theeke et al. 2017). In Nigeria where social security systems and health rehabilitation are generally lacking, establishing this support system will go a long way in the community reintegration of stroke survivors.

Managing the challenges of physical impairment constitute hindrances to community reintegration. Many complained of difficulty in moving or walking and thus, were limited in reintegration into the community. This has hindered them to participate in valued activities and roles. This resonates with other studies, which identified negative contextual factors to community reintegration (Chimatiro & Rhoda 2014; Govender et al. 2019; Van Dongen et al. 2021). Although the focus of rehabilitation is to improve functional outcomes, stroke survivors with severe functional deficits should be watched closely for possible assistance to aid functional recovery to facilitate community reintegration. Another barrier to community reintegration reported by our sample is the speech problem. Because of stroke survivors’ communication difficulties, they struggled to participate in meaningful activities they were involved in before the stroke (Manning et al. 2019). In addition to the impact of stroke, participation and autonomy are reduced because of aphasia (Manning et al. 2019). Therefore, rehabilitation interventions to support stroke survivors to build confidence in their communicative abilities and rebuild their sense of self are necessary (Wray & Clarke 2017). This is seriously lacking in Nigeria as very few hospitals have speech and language pathologists who should improve communication abilities post-stroke. There is a paucity of tertiary education facilities that train speech and language pathologists.

The outcome of our study may be restricted in external validity, considering that only 12 stroke survivors were interviewed. However, we ensured data saturation during participant recruitment and data collection. Our focus on qualitative methodology also limited the possibility of investigating variables that may describe the trends in community reintegration among stroke survivors; hence a more expansive method such as a mixed-method design may be useful for further investigations of determinants of community reintegration in this population subgroup.

In conclusion, we reported the experience of community integration among Nigerian stroke survivors. They had challenges returning to work and experienced varying levels of activity limitation, reflecting their QoL with identifiable enablers or barriers to community reintegration.
